# Nonadiabatic Tunneling in Chemical Reactions

**DOI:** 10.1021/acs.jpclett.4c01098

**Published:** 2024-07-12

**Authors:** Jeremy O. Richardson

**Affiliations:** Department of Chemistry and Applied Biosciences, ETH Zurich, 8093 Zurich, Switzerland

## Abstract

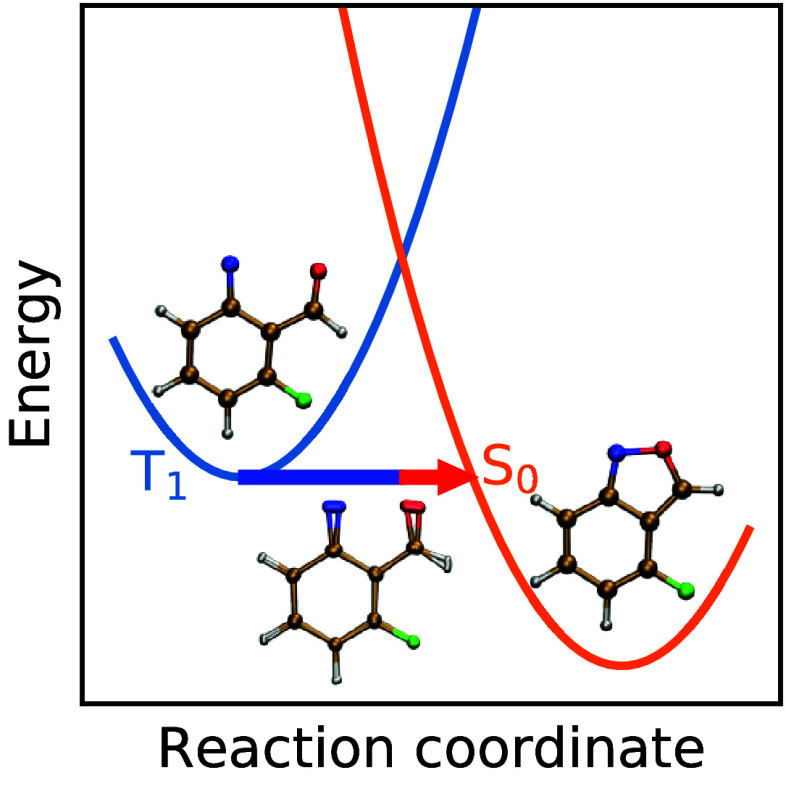

Quantum tunneling can have a dramatic effect on chemical reaction rates. In nonadiabatic reactions such as electron transfers or spin crossovers, nuclear tunneling effects can be even stronger than for adiabatic proton transfers. Ring-polymer instanton theory enables molecular simulations of tunneling in full dimensionality and has been shown to be far more reliable than commonly used separable approximations. First-principles instanton calculations predict significant nonadiabatic tunneling of heavy atoms even at room temperature and give excellent agreement with experimental measurements for the intersystem crossing of two nitrenes in cryogenic matrix isolation, the spin-forbidden relaxation of photoexcited thiophosgene in the gas phase, and singlet oxygen deactivation in water at ambient conditions. Finally, an outlook of further theoretical developments is discussed.

It is well-known that quantum tunneling of hydrogen atoms can affect the rate of chemical reactions at room temperature^1−3^ and even heavy-atom tunneling has been observed at low temperature.^4−8^ A number of theoretical approaches have been developed to describe and predict this effect, most notably semiclassical instanton theory,^9−13^ which has proved to be a powerful method even for large complex systems including water clusters,^14^ enzymatic reactions,^15^ homogeneous catalysis,^16,17^ as well as solid-state and surface processes.^18,19^

Although many of these chemical reactions and rearrangements are well described by the Born–Oppenheimer approximation, which constrains them to a single adiabatic state, there are other types of reactions which involve two or more electronic states and are therefore classified as “nonadiabatic” (see Figure 1). Typical examples include electron transfers^20^ and spin-crossover reactions (also known as intersystem crossing).^21^

**Figure 1:**
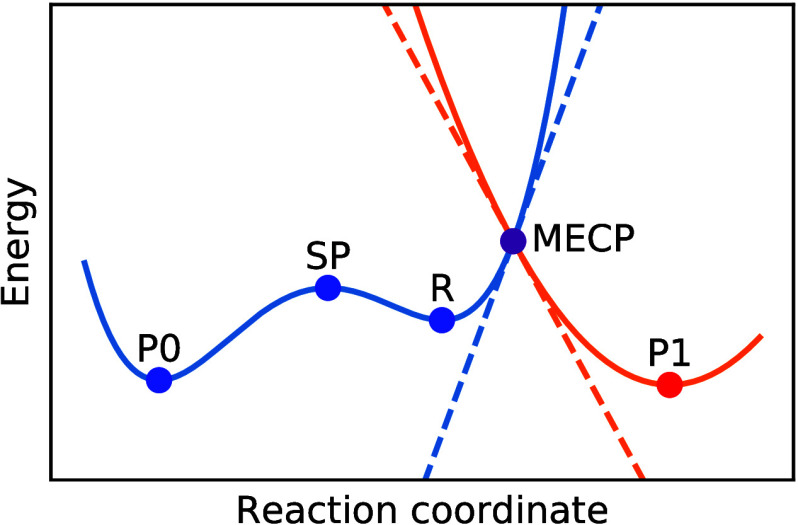
Schematic of a chemical reaction in a (spin-) diabatic representation. The reactants (R) can decay either via an adiabatic reaction through the saddle point (SP) to the products (P0) or via a nonadiabatic reaction (such as intersystem crossing) through the minimum-energy crossing point (MECP) to the products (P1).

Recently, we have derived a nonadiabatic instanton theory which describes tunneling in nonadiabatic chemical reactions.^22−24^ With this new approach, we have found that tunneling can be far more significant in nonadiabatic reactions than in typical adiabatic reactions and even allows for sizable heavy-atom tunneling effects at room temperature.

Before discussing instanton theory, it is useful to introduce the well established nonadiabatic rate theories that preceded it. Electron-transfer reactions are traditionally described using the classical picture of Marcus theory.^20^ Going beyond this, nonadiabatic transition-state theory, *k*_NA-TST_ ∝  provides a first-principles method which is commonly applied to calculate the rate of intersystem crossing.^25−27^ Here, the minimum-energy crossing point (MECP) with energy *V*^‡^ plays the role of the transition state and Landau–Zener transmission probabilities (proportional to Δ^2^) are employed to account for nonadiabatic effects. Although NA-TST takes account of zero-point energy, it does not have tunneling effects built in.

Tunneling corrections to NA-TST have been proposed based on one-dimensional pictures assuming the separability of the reaction coordinate from all other degrees of freedom.^28^ For instance, the weak-coupling (WC) method uses quantum-mechanical perturbation theory to determine the transmission probability.^29^ However, in order to simplify the computation, it approximates the potentials as linear functions (shown in Figure 1 as dashed lines), for which the exact solution of the Schrödinger equation is known. This linear approximation is valid only at high temperatures where the tunneling occurs close to the MECP, but as soon as deep tunneling sets in, the approximation that the potentials are linear breaks down and it can lead to orders of magnitude of error and a failure to describe the correct low-temperature plateau of the rate. Note that although one can use the Zhu–Nakamura (ZN) approach^30^ to go beyond perturbation theory, this does not fix the main problem as it is still based on one-dimensional linear potentials.

There are, however, a number of ways to go beyond the linear approximation using semiclassical techniques.^31−39^ Of these, nonadiabatic instanton theory is unique in providing a practical tool for first-principles calculations of molecular tunneling in full dimensionality.^23,40^ In particular, it locates the tunneling pathway directly on the ab initio potential-energy surfaces. Instead of taking *global* approximations, instanton theory performs a *local* approximation around this pathway, making it applicable to any problem with a well-defined local environment, such as gas-phase molecules, or those trapped in solids or on surfaces.

## Derivation of Instanton Theory

In many cases of interest, there are only two relevant diabatic electronic states σ = 0 and σ = 1 corresponding to the reactant and product with potential-energy surfaces *V*_σ_(*x*) and a coupling Δ(*x*). For example, in an electron-transfer reaction, the two states are localized on the donor or acceptor, whereas in a spin-crossover reaction, the electronic states are distinguished by their different spin quantum numbers and are coupled by spin–orbit coupling. For a system with *f* nuclear degrees of freedom with coordinates *x* = {*x*_1_, ..., *x*_*f*_} associated with momentum operators *p̂*_α_ and masses *m*_α_, the Hamiltonian corresponding to each state is *Ĥ*_σ_ = *T̂*_n_ + *V*_σ_(*x*), where the nuclear kinetic energy is *T̂*_n_ = ∑_α_*p̂*_α_^2^/2*m*_α_.

**Figure 2:**
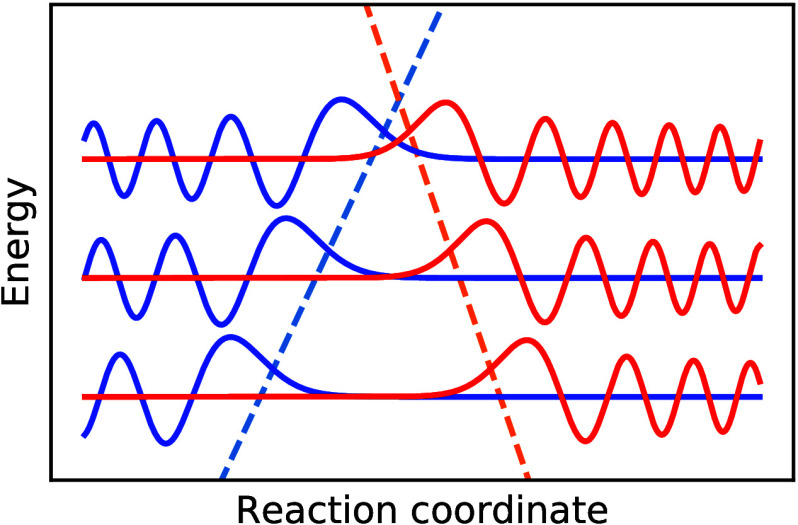
Example of FGR for a system of two linear potentials. The square of the overlap  between the reactant and product wave functions of equal energy determines the tunneling probability; it is large close to the MECP and decreases at lower energies.

In many nonadiabatic reactions, we can assume that Δ is small; this is typical for electron-transfer reactions with a large distance between donor and acceptor^36^ as well as for intersystem crossing because of the small magnitude of spin–orbit couplings.^21^ In these cases, we can make use of second-order perturbation theory^41^ to write the thermal rate constant, *k*, at reduced temperature β =  as^a^
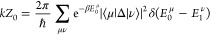
1where the (ro)vibrational eigenstates (stationary nuclear wave functions) obey *Ĥ*_0_|μ⟩ = *E*_0_^μ^|μ⟩ and *Ĥ*_1_|ν⟩ = *E*_1_^ν^|ν⟩, and the reactant partition function is *Z*_0_ = ∑_μ_e^–*βE*_0_^μ^^.^b^ This formulation is known as Fermi’s golden rule (FGR),^42^ and in order to implement it directly, one requires complete knowledge of the (ro)vibrational states of *Ĥ*_0_ and *Ĥ*_1_ (Figure 2). Although it is true that this is not as hard as finding the eigenstates of the full coupled problem, it is still intractable for a complex molecule unless global harmonic approximations are employed.^21^ We will thus search for an alternative formulation of FGR which does not involve the nuclear wave functions at all. In this way, we will employ perturbation theory without having solved the Schrödinger equation for the unperturbed problem.

This can be achieved in the following manner. First, replace e^–*βE*_0_^μ^^ in eq 1 by e^–(β–τ/*ℏ*)*E*_0_^μ^^ e^–*τ E*_1_^ν^/*ℏ*^, which is valid for arbitrary τ ∈ [0, *βℏ*] as the delta function ensures energy conservation from the initial to final state. Second, use the Fourier representation of the delta function,^41^ δ(*E*_0_^μ^ – *E*_1_^ν^) = . Third, use the eigenvalue relations to replace *E*_0_^μ^ by *Ĥ*_0_ and *E*_1_^ν^ by *Ĥ*_1_. Finally, remove the sums over states using the resolution of the identity 1 = ∑_μ_ |μ⟩⟨μ| = ∑_ν_|ν⟩⟨ν|.

This leaves us with an exact reformulation of FGR:^43,44^
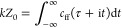
2a
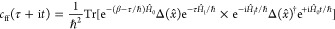
2bwhere the trace is performed over the nuclear degrees of freedom only. What we have achieved in reformulating eq 1 in this way is that the trace in eq 2b is basis-independent and the eigenstates do not appear, meaning that one does not have to explicitly solve the (ro)vibrational Schrödinger equation. We note that these formulas can alternatively be derived starting from Miller’s flux correlation function^45^ and taking the weak-coupling limit using time-dependent perturbation theory.^46,47^ The expressions are formally applicable to complex multidimensional systems, although calculating eq 2b exactly is still an impossibly hard problem in most cases. Its main advantage is that it is a good starting point for developing approximations, as we shall show.

In particular, we shall demonstrate how good approximations to *c*_ff_(τ) can be made, i.e., for the case of *t* = 0. To do this, we insert the identity 1 = ∫_–*∞*_^*∞*^ |*x*⟩⟨*x*| d*x* twice (once with dummy variable *x′* and once with *x*″) to obtain
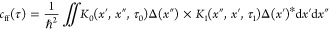
3where we have defined τ_0_ ≡ *βℏ* – τ and τ_1_ ≡ τ. The propagator *K*_σ_(*x′*, *x*″, τ_σ_) = ⟨*x′*|e^–τ_σ_*Ĥ*_σ_/*ℏ*^|*x*″⟩ is the probability amplitude for the molecule to rearrange from configuration *x′* to *x*″ in imaginary time −iτ_σ_. Note that the imaginary-time propagator is mathematically equivalent to the thermal density matrix with a temperature of *ℏ*/*k*_B_τ_σ_.

The propagators can be alternatively defined using the path-integral formulation of quantum mechanics,^48^*K*_σ_(*x*′, *x*″, τ_σ_) = . In this theory, all possible paths which start at *x′* and end at *x*″ contribute. Each path is weighted according to the (Euclidean) action, *S*_σ_ = ∫_0_^τ_σ_^ [*T*_n_(*ẋ*(*u*)) + *V*_σ_(*x*(*u*))]d*u*, where the kinetic energy  and imaginary velocity  are defined at each imaginary time *u* ∈ [0, τ_σ_] along the path *x*(*u*).

It is at this point that we can introduce our first approximation to FGR. Because of the behavior of the exponential function, the path integral is clearly dominated by paths with the smallest values of *S*_σ_. One thus expects that a good approximation to the propagator can be found in which knowledge only of the immediate vicinity of the minimum-action pathway is necessary. A careful analysis shows that this is indeed true,^49^ and one obtains the semiclassical approximation *K*_σ_(*x*′, *x*″, τ_σ_) ≃, evaluated along the trajectory which minimizes the action. This is an asymptotic approximation which becomes exact in the *ℏ* → 0 limit. We call the minimum-action pathway a trajectory because it obeys the principle of least action and hence the Euler–Lagrange equations of classical mechanics.^50^ However, it is not an ordinary classical trajectory because it moves in imaginary time. It thus follows a modified version of Newton’s second law, *F* = −*ma* (where *F* = –*∂V*_σ_/*∂x* is the force and *a* = *ẍ* the acceleration), and can be equivalently thought of as a Newtonian trajectory in the upside-down potential.^51^ The energy *E*_σ_ = (*∂S*_σ_/*∂τ*_σ_)_*x′*,*x*″_ = *V*_σ_(*x*(*u*)) – *T*_n_(*ẋ*(*u*)) is conserved along the trajectory. This then provides a simple extension of classical mechanics which enables trajectories to tunnel through barriers with *E*_σ_ < *V*_σ_(*x*).^10^ The prefactor *C*_σ_ accounts for fluctuations to second order around the trajectory and encodes local harmonic zero-point energy effects. It can be constructed from the Hessians along the path^11^ and is a generalization of the van-Vleck–Gutzwiller propagator^52^ to imaginary time.

After inserting the semiclassical propagators into eq 3, we note that the integrand includes the factor e^–*S*/*ℏ*^, where *S* is the total action *S*(*x′*, *x*″, τ) = *S*_0_(*x′*, *x*″, *βℏ* – τ) + *S*_1_(*x*″, *x′*, τ). Therefore, in order to perform the integrals over *x′* and *x*″ for a given value of τ, we can use the method of steepest descent,^53^ i.e., locate the minimum of *S* and expand it as a Taylor series truncated after second order. Neglecting the slowly varying prefactors, the integral is then just a multidimensional Gaussian, which can be evaluated analytically to give
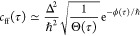
4where ϕ(τ) is the action *S*(*x′*, *x*″, τ) at the τ-dependent optimal positions of *x′* and *x*″ (i.e., those which minimize *S*). The total fluctuation factor is determined by Θ(τ) = *C*(τ)/[*C*_0_(τ_0_)×*C*_1_(τ_1_)], where *C*(τ) is the determinant of a matrix of second derivatives of *S* with respect to *x′* and *x*″.^22^ Finally, Δ^2^ = Δ(*x′*)*Δ(*x*″) is evaluated at the hopping points where the change in electronic state takes place.^c^

After all the formal mathematics, we now have a computationally feasible approach which approximates the flux correlation function at a given value of τ. In contrast to the fully quantum approach, all we need is the optimal tunneling pathway, which travels as a classical trajectory in the upside-down reactant potential from *x′* to *x*″ in imaginary time *βℏ* – τ, changes electronic state, continues on the upside-down product potential from *x*″ to *x′* in imaginary time τ, and returns to its original state. The total imaginary time elapsed is thus *βℏ*. The hopping points *x′* and *x*″ are determined by locating a stationary point of *S*(*x′*, *x*″, τ). At the stationary point, we have  = 0, which (using results from classical mechanics)^50^ implies that the momentum changes continuously across the hops.^22^ The two trajectories thus combine to form a periodic orbit, i.e., a closed path in phase space (Figure 4b*′*). This periodic orbit describes the optimal tunneling pathway of the molecular system, and we call it an “instanton” due to its mathematical similarity with the instantons of quantum field theory.^54^ In gas-phase reactions, there will typically be a unique instanton pathway, whereas on surfaces, there may be multiple stationary-action solutions due to different local environments^19,55^ which should be considered separately.^d^

**Figure 3:**
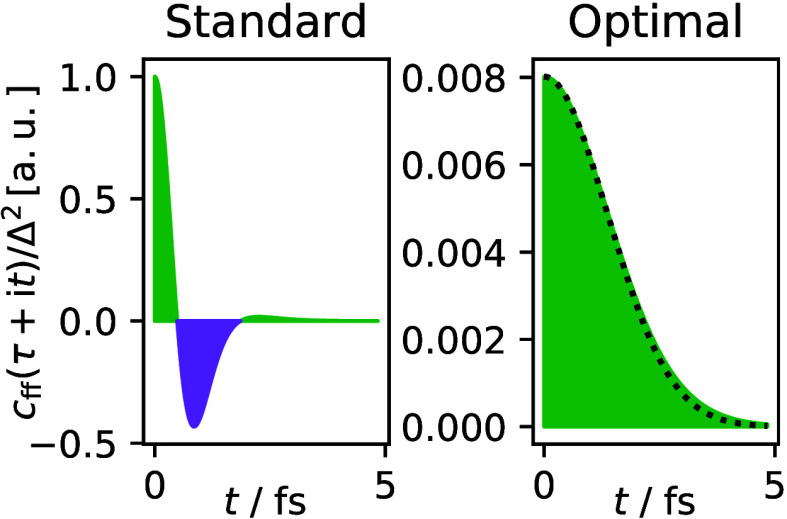
Flux correlation function for a typical example of electron transfer defined by a symmetric spin-boson model at room temperature with a reorganization energy of 50 kcal/mol parametrized by an Ohmic spectral density with an exponential cutoff frequency of 3000 cm^–1^. The left panel shows the standard form with τ = 0, whereas the right panel uses the optimal value (τ = *βℏ*/2 in this case). Due to large cancellations between positive and negative lobes, the former integrates to give the same rate as the latter, but only with the optimal choice is the integrand approximately a Gaussian (dotted line). Note that we have only shown the real part at positive times as it is a Hermitian function.

**Figure 4:**
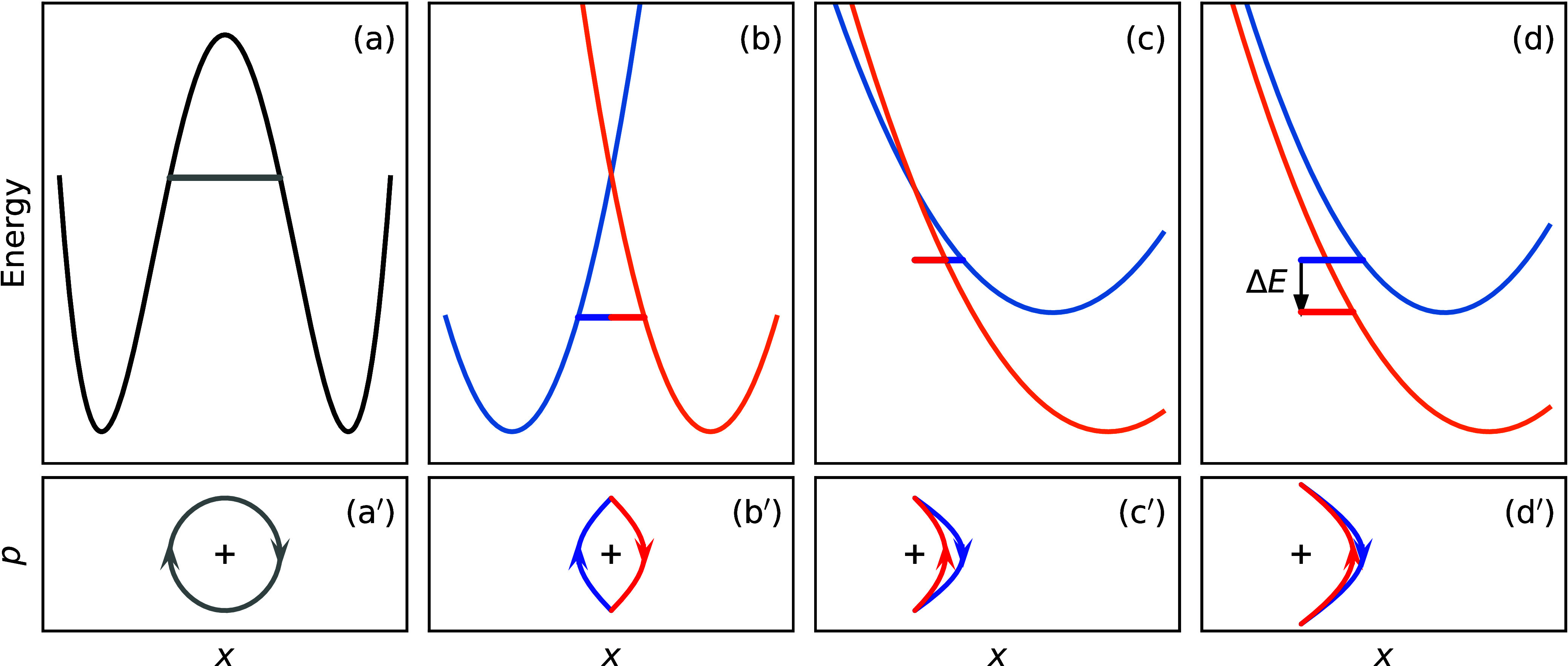
Four types of tunneling pathways for schematic one-dimensional models of (a) an adiabatic reaction; (b) a nonadiabatic reaction in the normal regime; (c) a stationary-action instanton in the inverted regime; (d) a branch-point instanton. Panels a*′*–d*′* show the phase-space representation of the corresponding periodic orbits in the upside-down potentials with the saddle point or hopping point indicated by a cross (at *p* = 0). In the three nonadiabatic cases, blue corresponds to the reactant σ = 0 and red/orange to the product σ = 1.

Finally, to obtain a prediction for the rate, we must carry out the time integral in eq 2a.^e^ As is clear from our derivation above, the integral over the flux correlation function formally gives the correct result, regardless of the value of τ (at least within the range [0, *βℏ*]). However, as demonstrated in Figure 3, even though the value of the integral is the same, the correlation function itself depends strongly on the choice of τ. We will not be able to reliably integrate a complicated function, as through the semiclassical instanton approach, we only have access to information about *c*_ff_(τ) at *t* = 0. The principle of steepest-descent integration^53^ again offers a solution by approximating the correlation function by a Gaussian around the optimal value of τ. In the standard case, the optimal value of τ is a maximum of ϕ(τ) and thus obeys  and ϕ″(τ) < 0 and is therefore known as the stationary-action point, τ_SA_. We then use a Taylor series to expand the action to second order, , where ϕ_SA_ = ϕ(τ_SA_) and we have used the Cauchy–Riemann relation .^57^ In order to obtain the leading-order term, it is only necessary to expand the prefactors to zeroth order, , and similarly for Δ^2^, which is formally τ-dependent through its dependence on *x′* and *x*″. This gives
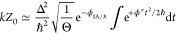
5a
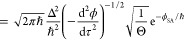
5bAll terms are evaluated for the instanton which is defined at the stationary point with respect to *x′*, *x*″ and τ. Because of the stationary-action requirement, , the energy of both trajectories which make up the instanton are equal, *E*_0_ = *E*_1_, which would not be the case for arbitrary τ. This is the semiclassical equivalent of the delta function which appears in eq 1 and ensures that the total energy is conserved in passing from reactants to products. A typical instanton pathway is shown in Figure 4b. Together the momentum constraint and the energy constraint force the hops to occur on the crossing seam,^22,24^ although not necessarily at the MECP, due to corner cutting.

Let us now interpret the formula in physical terms. The most obvious observation is that the rate is proportional to Δ^2^, due to the second-order perturbative expansion of all golden-rule theories. More interestingly, instead of the usual Arrhenius factor e^–*βV*^‡^^ of NA-TST, the rate is dominated by the exponential term e^–ϕ/*ℏ*^, which can be decomposed into a tunneling probability e^–*W*/*ℏ*^ and a Boltzmann factor e^–*βE*^, using the Legendre transform ϕ = *W* + *βℏE*,^10^ where *E* = *E*_0_ = *E*_1_ is the energy of the instanton. There is a simple geometric interpretation for *W* = ∮*p*·d*x* as the phase-space area enclosed by the periodic orbit (Figure 4b*′*). The semiclassical approach thus captures the same behavior observed in Figure 2 where the tunneling probability increases with energy. In contrast, the Boltzmann factor decreases with energy, and in general, the stationary-action instanton finds a compromise at an intermediate energy. However, in the low-temperature limit, the Boltzmann factor dominates and the instanton energy approaches that of the reactant minimum such that the rate, which is dominated by e^–*W*/*ℏ*^, becomes independent of temperature. In the high-temperature limit, the instanton collapses at the MECP where the tunneling probability is highest; that is, *W* shrinks to 0 and *E* tends to *V*^‡^ such that the theory reduces to NA-TST.^22,40^ In this way, we see that it is the whole instanton path that plays the role of the “transition state” of a tunneling process.

It is interesting to compare the golden-rule instanton result with the Born–Oppenheimer limit. In an adiabatic reaction, the barrier typically has a parabolic shape, *V*(*x*) =  at its top (Figure 4a). Because the sharp cusp of the nonadiabatic case is narrower than a parabola (at least close to the barrier top),^f^ the area enclosed by the nonadiabatic periodic orbit will be smaller (Figure 4b*′* vs Figure 4a*′*) leading to a higher tunneling probability.

In the adiabatic case, the upside-down potential is an oscillator, which cannot support periodic orbits with an arbitrarily short period. This means that there is a crossover temperature  above which no instantons can exist. In the golden-rule case, the potentials meet at a cusp (Figure 4b), and thus, periodic orbits of any period can be supported, which implies that instantons exist at all temperatures. As a simple example, we can take the model of two crossed linear potentials, *V*_σ_ = *V*^‡^ + κ_σ_*x* (Figure 2), for which eq 5b can be evaluated analytically. In this special case, we can additionally show that the steepest-descent integrations are exact and thus the instanton result is identical to the quantum-mechanical FGR.^22^ In this case, the tunneling factor, defined as the ratio between the instanton rate and its classical limit, is , where  is the temperature of the reaction in Kelvin and
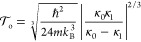
6is called the “onset temperature”.^58^ This provides a useful measure of when tunneling is expected to be important and can be evaluated relatively easily as it requires data obtained from the MECP only (in particular the signed norm of the mass-weighted gradients ). When , tunneling has little effect on the rate, whereas when , tunneling is expected to be significant. In the latter case, it is not advised to trust the simple tunneling factor derived for the linear model for quantitative results and a full instanton calculation should be initiated.

Up until now, we have considered peaked crossings (as in the Marcus normal regime, Figure 4b), but remarkably the instanton approach continues to be applicable also for sloped crossings (as in the Marcus inverted regime, Figure 4c).^24^ In order to form a periodic orbit in Figure 4c*′*, it is necessary for the trajectory to perform a dramatic change of direction after the hops between reactant and product states. This has a number of interesting consequences. First, a significantly smaller area of phase space is enclosed by the orbit, making the tunneling probability e^–*W*/*ℏ*^ much higher than in the normal regime. Second, we note that in phase space, particles always flow forward in a clockwise fashion. Therefore, in order to move in an anticlockwise fashion on the product state in Figure 4c*′*, it is necessary to move backward in imaginary time, implying that τ must be negative. This means that the propagator *K*_1_ becomes undefined as it would be equivalent to density matrix with an unphysical negative temperature *ℏ*/*k*_B_τ.^46,59^ According to the Boltzmann distribution, negative temperatures favor high-energy states and, as the Hamiltonian is unbounded from above, will lead to a divergence. Nonetheless, the path-integral formalism can be rigorously extended to treat this case.^40^ In particular, the instanton method is saved by the fact that at a stationary point of *S*, the energy of reactants and products are constrained to be equal. The instanton thus finds a compromise between its desire to lower the energy of the reactants (due to their positive temperature) and its desire to raise the energy of the products (due to their negative temperature), and in this way it avoids any divergences.

**Figure 5:**
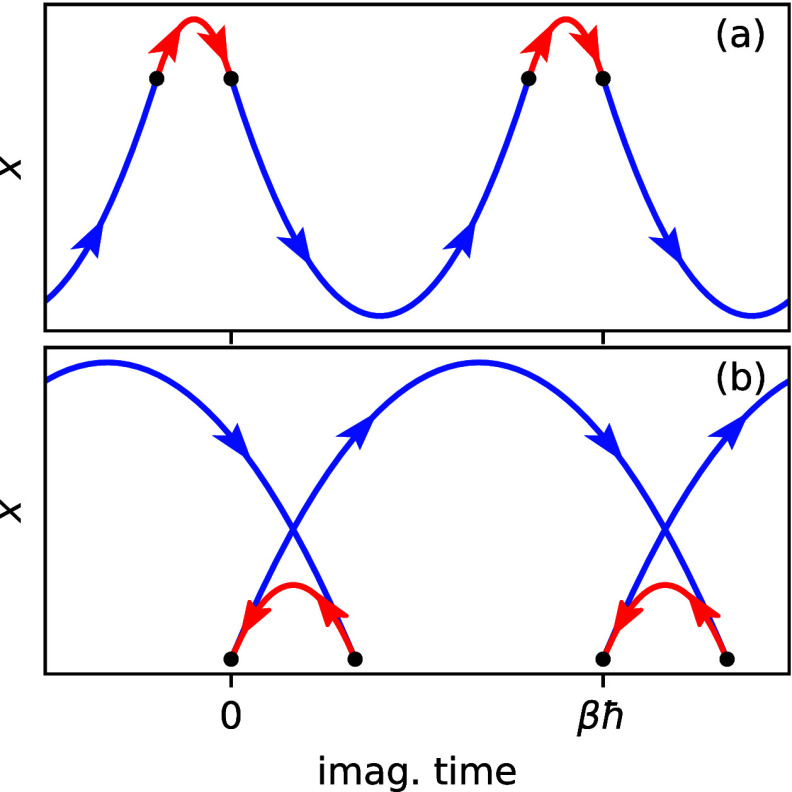
Golden-rule instanton trajectories for (a) the normal regime and (b) the inverted regime. In both cases, the orbit is periodic with period *βℏ*.

A complementary picture of the instanton pathways is presented in Figure 5, in which the nuclear configuration is plotted against imaginary time along the trajectory. In the normal regime, the periodic orbit follows straightforward bouncing trajectories with hops between the reactant and product states. However, the picture in the inverted regime is complicated by the negative time propagation. In particle physics, it is common to identify objects moving backward in time as antiparticles.^60^ Reading Figure 5b chronologically from left to right, we thus obtain a new interpretation of the inverted-regime tunneling pathway as follows: we start with a molecule tunneling in imaginary time on the reactant state; at some point a molecule/antimolecule pair is created; the new molecule is also associated with the reactant, but the antimolecule moves on the product state until it meets with the original molecule, which annihilate each other leaving only the new molecule. The idea that chemical reactions involve antiparticle versions of molecules sounds crazy at first sight. However, note that the antimolecules only exist in imaginary time and are thus not experimentally observable. One might ask whether something which cannot be observed experimentally can be said to be “real” at all. This is a question for philosophers to debate. Instead, we simply argue that the interpretation in terms of antimolecules is no less valid than the usual FGR approach based on wave function overlaps, remembering of course that wave functions are also not experimental observables. However, in contrast to the wave-function approach, the instanton-based antiparticle picture leads to a simple mechanistic interpretation as well as reliable predictions obtained from computationally viable methodology, as we shall demonstrate later for the example of thiophosgene.

**Figure 6:**
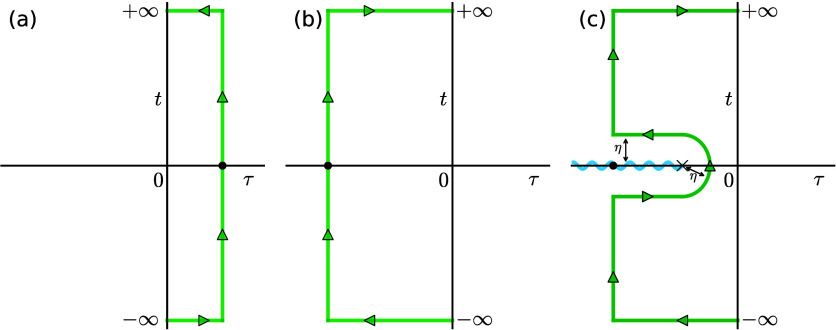
Integration contours in the complex-time plane for (a) the normal regime, (b) the inverted regime, and (c) the case with a branch point. The black circle indicates the stationary-action point, τ_SA_; the cross indicates the branch point, τ_BP_; and the wavy line indicates the branch cut.

Mathematically speaking, the procedure that was used to obtain the instanton theory in the inverted regime involves deforming the contour of integration in eq 2a. Cauchy’s theorem states that all integration contours give the same result as long as they can be deformed into each other without crossing any singular points.^57^ It is clear from the derivation above that *c*_ff_ has no singularities in the interval τ ∈ [0, *βℏ*], such that the normal regime requires no special treatment (Figure 6a). It is also possible to show that for a spin-boson model *c*_ff_ is analytic everywhere in the complex plane and therefore cannot have any singular points, even in the inverted regime.^24^ In many cases, therefore, one can deform the contour as shown in Figure 6b. However, this will not be true in general and even a harmonic model in which the frequencies of the reactant and product states differ can have singularities where Θ(τ) = 0 for τ < 0. This causes no problem to the theory presented above if the roots are to the left of the stationary-action point, τ_SA_, but if they appear to its right, we must modify the approach. Because Θ(τ) appears within a square-root function, these points are “branch points” and are associated with a branch cut that extends from the branch point to complex infinity. In order to obey Cauchy’s theorem, we must deform the contour to avoid the branch cut and go around the branch point. An appropriate choice is depicted in Figure 6c.

Assuming that there is only one relevant branch point at τ = τ_BP_ > τ_SA_, we follow the steepest-descent procedure along the new contour to find^61^


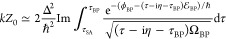
7a
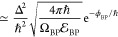
7bwhere Ω_BP_ = Θ*′*(τ_BP_), ϕ_BP_ = ϕ(τ_BP_), . To obtain the expression in the final line, we have taken the limit η → 0_+_ and assumed that the stationary-action point is so far to the left (i.e., τ_SA_ ≪ τ_BP_) that we can effectively replace it by −∞.

A branch point will exist in the instanton approximation to the correlation function whenever the determinant of the Hessian is zero, i.e., *C*(τ) = 0, which implies that a zero-frequency mode appears as a manifestation of an infinite family of instantons with identical actions.^g^ At the branch point, there is no requirement that the energies of the two classical trajectories match, such that in general Δ*E* = *E*_0_ – *E*_1_ = *V*_0_ – *V*_1_ ≠ 0 (Figure 4d) and the hop must occur away from the crossing seam.

When the semiclassical rate is given by eq 7a, it is no longer dominated by the stationary-action point but instead has an exponential dependence on the action at the branch point. As the stationary-action point was a maximum, the action at the branch point is by definition smaller (as is also seen from the region of phase space enclosed in Figure 4d*′*), which makes the rate significantly larger. In addition, the instanton energy *E* =  can be much lower than the MECP energy. Together, these two effects can lead to enormous tunneling factors as we shall demonstrate with the case of singlet oxygen deactivation.

## Applications

The ring-polymer formulation allows nonadiabatic instanton theory to be applied to molecular systems using standard quantum-chemistry approaches,^40^ similar to that used for adiabatic tunneling calculations.^11^ In this method, the path is discretized using *N* = *N*_0_ + *N*_1_ replicas of the molecular geometry, called beads, {*x*^(1)^, ..., *x*^(*N*)^}. These beads determine the start and end of *N* short straight-line segments, of which *N*_0_ describe the reactant trajectory and *N*_1_ the product trajectory. The number of beads should be increased until the results converge to a required precision. The action as a function of the discretized path becomes
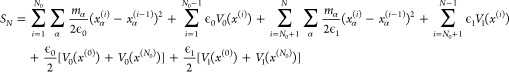
8where ϵ_0_ = (*βℏ* – τ)/*N*_0_ and ϵ_1_ = τ/*N*_1_ are the imaginary times of each segment and the cyclic boundary conditions impose that *x*^(0)^ ≡ *x*^(*N*)^. The stationary-action instanton is defined as the stationary point of *S*_*N*_ with respect to all beads and τ simultaneously. In the normal regime, the instanton is saddle point with index one, whereas in the inverted regime it has an index of *N*_1_*f* + 1.^24^ The saddle points are found using quasi-Newton optimization methods,^62^ which typically converge quickly and reliably especially when using a good initial guess based on a previous instanton optimization with fewer beads or at a higher temperature. In most cases, the path is found to fold back on itself and so a factor of 2 time-saving can be made by equating pairs of beads.^40^ Zero frequencies corresponding to translational and rotational modes of the whole ring polymer are removed in the usual way before evaluating Θ, defined as the determinant of the Hessian of *S*_*N*_.^40,63^ To find branch points, one optimizes the beads for a set of fixed values of τ to determine ϕ(τ) = *S*_*N*_ and Θ(τ). An interpolation gives the required root of Θ and the necessary derivatives.^61^

There is only one remaining part of the algorithm that we need to discuss: a method is required to evaluate the diabatic potentials *V*_σ_(*x*) at arbitrary geometries. Nonadiabatic instanton theory is thus very well suited to studying spin-crossover reactions as standard quantum-chemistry packages return the appropriate spin-diabatic states, e.g., for the singlet or triplet potentials. In this way, the ring-polymer optimization is easily coupled to existing electronic-structure codes, making use of analytic gradients where available. Note that the spin–orbit coupling, Δ, only needs to be computed once, after the instanton is located.

**Figure 7:**
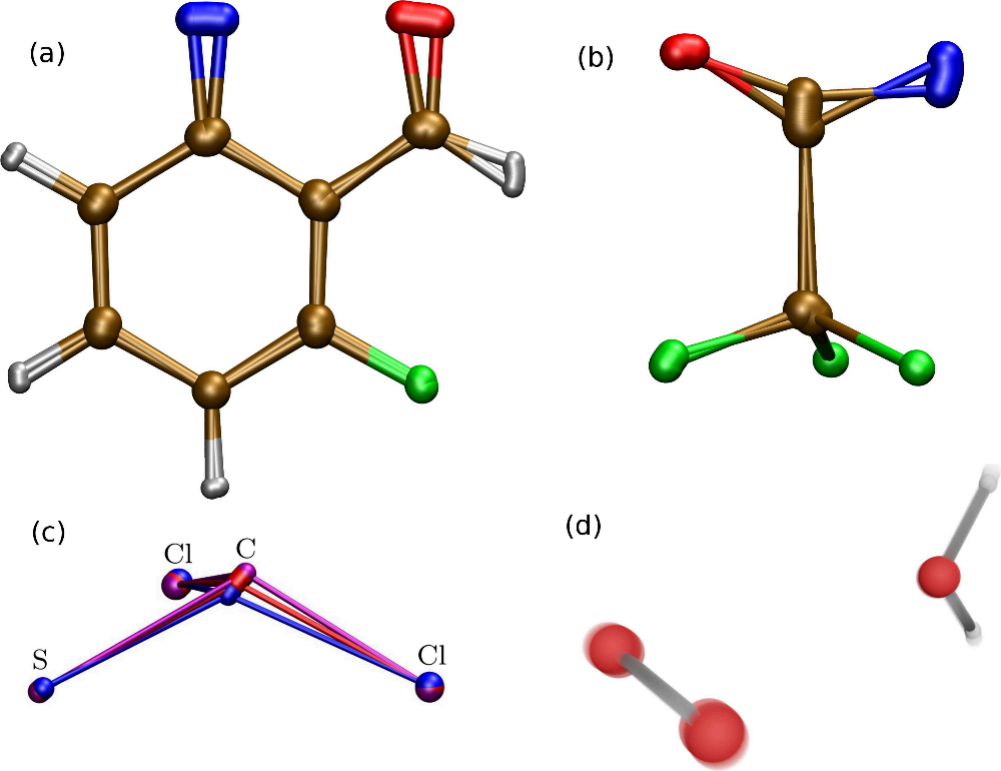
Ab initio ring-polymer instantons for (a) cyclization of C_7_H_4_ONF, (b) isomerization of C_2_ONF_3_, (c) thiophosgene (blue/red for reactant/product), and (d) ^1^O_2_ deactivation in D_2_O. Adapted from refs (58, 66), and (61).

Nitrenes provide an interesting chemical playground for spin-crossover reactions. It is possible to prepare triplet nitrenes in inert-gas matrices at low temperatures and to experimentally measure the rate of decay to the singlet state, which is accompanied by a molecular rearrangement, by probing the evolution of the vibrational frequencies. In the two molecules measured in refs (64) and (65), the rate was seen to plateau at low temperature, a signature of deep tunneling. Previous attempts to calculate the rate using the WC method could neither quantitatively reproduce the experimental results nor even predict that a low-temperature plateau exists.^64^ We optimized instantons with the relatively cheap double-hybrid density-functional theory (DFT) as shown in Figure 7a,b.^58^ The rate predicted at this level was an order of magnitude too large. However, by correcting the energies with the more expensive multireference Møller–Plesset (MRMP2) method, instanton theory was able to reproduce the experimental results to a high accuracy including the correct physical behavior in the low-temperature limit (Table 1). Additionally a large ^14^N/^15^N kinetic isotope effect (KIE) due to heavy-atom tunneling of the nitrogen atom was reproduced. We also made predictions of even larger KIEs for C or O substitutions,^58^ which we hope to be confirmed by future experiments.

**Table 1:** ^14^N/^15^N Kinetic Isotope Effects (KIEs) Are Reported for the Two Nitrenes, ^12^C/^13^C KIEs for Thiophosgene, and H_2_O/D_2_O for ^1^O_2_ in Water^a^

				*k*/s^–1^		KIE	
Reaction				SCI	Expt.	SCI	Expt.
nitrene cyclization	434	300	4	1.1 × 10^4^	–	1.0	–
		low	–	1.2 × 10^–3^	1.4 × 10^–3^	1.4	–
nitrene isomerziation	514	300	60	1.3 × 10^1^	–	1.04	–
		low	–	1.8 × 10^–3^	1.2 × 10^–3^	1.35	1.18–1.44
thiophosgene	696	300	600	8.5 × 10^7^	–	1.4	–
		ZPE	–	4.5 × 10^7^	4.4 × 10^7^	2.3	–
O_2_ in water	2300	300	10^27^	≈1.7 × 10^5^	2.9 × 10^5^	21.0	19.7

a is the molecule-specific onset temperature. The temperature used for the experiment/calculation is given by  unless it was in the low-temperature limit or state-selected to the zero-point energy (ZPE). The tunneling factor is the ratio between the semiclassical instanton (SCI) rate and the NA-TST rate and does not have a well-defined low-temperature limit as the latter tends to zero.

Whereas the two nitrene reactions took place in the normal regime, the triplet–singlet transition in thiophosgene, CSCl_2_, is in the inverted regime. Spectroscopic experiments have been performed in the gas phase in which the lifetime of specific vibrational states of the triplet were measured.^67^ Again previous theoretical attempts based on the WC or ZN methods had predicted that tunneling was involved but had failed to obtain quantitative agreement.^68^ Because thiophosgene is a reasonably small molecule, we were able to run instanton calculations with on-the-fly MRMP2 calculations.^66^ No evidence of branch points was found. Nonetheless, as expected from the high onset temperature, we predict a large tunneling factor of about 600 at room temperature involving heavy-atom tunneling of the carbon atom (Figure 7c).^h^ By converting the thermal rates into microcanonical rates using an approximation to the inverse Laplace transform,^69−72^ we obtained the rate of decay from the ground vibrational state of the triplet shown in Table 1, which is again in remarkable agreement with experiment. To our knowledge, the ^12^C/^13^C KIE has not been measured for thiophosgene. We provide a tantalizing prediction of an unprecedentedly large value of 2.3 (considering that only heavy atoms are involved) to encourage experiments to be carried out.

Our final example is singlet oxygen deactivation, an important process with both chemical and biological significance as well as a long history of scientific studies. Recently, careful experiments have revealed interesting solvent-dependent and temperature-dependent lifetimes, including a large H_2_O/D_2_O KIE of about 20 for the nonradiative relaxation of ^1^O_2_ to ^3^O_2_ in liquid water.^73^ For simplicity (and following previous theoretical work)^74^ we studied a 1:1 complex of O_2_···H_2_O and its deuterated variant using the multiconfigurational self-consistent-field (MCSCF) approach (Figure 7d). The singlet–triplet transition occurs in the inverted regime and exhibits a branch point at a value of τ_BP_ > τ_SA_. The branch-point instanton has tunneling contributions from the O_2_ stretch as well as the symmetric stretch of the water molecule. The rate calculation suggests that the speed-up due to tunneling is on the order of 10^27^, which is surely one of the largest tunneling effects reported for a chemical reaction at room temperature. It changes the lifetime from longer than the age of the universe to a few microseconds. This result may appear unbelievable, but it can be rationalized from the dramatic change in reaction mechanism predicted by the branch-point instanton instead of the classical mechanism through the MECP, which is particularly high in energy in this case. It is difficult to directly calculate the lifetime measured by experiment without knowing the equilibrium constant for complexation. However, a rough estimate combined with the instanton calculation of the rate constant gives reasonable agreement with experimental measurements as shown in Table 1. An even better test is provided by the KIE where the effect of the equilibrium constant is expected to cancel out. Here, the theory is in excellent agreement, which justifies our mechanistic interpretation.

## Discussion

In the past, tunneling effects have often been neglected in chemical reaction rate theory. It has been assumed that unless one is at very low temperature, heavy-atom tunneling is negligible, and that although hydrogen atoms may contribute to a small tunneling effect at room temperature, this can be treated well using simple approximations. This wisdom is based on accumulated experience with adiabatic reactions. However, it seems that nonadiabatic reactions offer a completely new and exciting world in which heavy-atom tunneling can play a significant role even at room temperature, and where the effects of corner cutting and deep tunneling are so pronounced that they require more advanced methods like instanton theory for a reliable treatment. The simple reason for this is that adiabatic barriers have a rounded top, whereas nonadiabatic crossings have cusped or sloped potentials. Because the width of the barrier is such an important factor in determining the tunneling probability, the wide, rounded top does not enable heavy-atom tunneling at room temperature, whereas the sharp, narrow nonadiabatic crossings do. The sloped crossing (inverted regime) has even more pronounced tunneling effects than the cusped crossing (normal regime) because of the negative imaginary time, which leads to a negative value for *S*_1_ and hence a smaller total *S*. In some cases, the existence of branch points enables the dominant instanton to break the usual energy-matching condition, which further reduces the action and leads to dramatic increases of the tunneling rate of many orders of magnitude. It is not yet clear whether the existence of branch points will be rare or common in inverted-regime reactions. However, branch points will definitely occur in extreme cases where the two surfaces do not cross at all, such that the classical process is forbidden and tunneling is the only possible reaction mechanism.

At first sight, the picture of nonadiabatic tunneling offered by instanton theory seems quite different from more conventional approaches such as Marcus–Levich–Jortner theory^36^ and its extensions for proton-coupled electron transfer,^75^ which describe tunneling using a sum over Franck–Condon factors calculated between the quantized states of a subset of high-frequency modes. Nonetheless, we have shown that instanton theory gives similar predictions and in many cases a more intuitive mechanism.^24,56^ Importantly, the instanton method does not require the wave functions and can thus be applied to systems with a large number of coupled vibrational modes. Unlike the cumulant expansion (which does not obey detailed balance in general), it remains accurate even when these modes are anharmonic^56^ and for asymmetric system–bath models where the reorganization energy of the reactants does not match the reorganization energy of the products.^76^

One of the key reasons for the success of the instanton approach is that it retains the rigor of quantum-mechanical path-integral theories without incurring sign problems or even the computational cost of thermodynamic sampling. Instead of calculating the contributions of many paths, we focus only on one. This instanton trajectory has imaginary-time length *βℏ*, which at room temperature corresponds to about 25 fs and grows to 250 fs at 30 K. The number of beads, *N*, required for convergence is thus on the order of 100 or 1000, and the bottleneck is the evaluation of the ab initio potentials at each bead. The computational expense is not only much less than that of full quantum dynamics methods, which typically require global potential-energy surfaces, but it is also much more efficient than path-integral Monte Carlo methods and even ab initio molecular dynamics simulations. Although it is true that the instanton calculation additionally requires Hessian (second derivative) information along the pathway, this can be easily parallelized and made even more efficient using interpolation or machine-learning techniques, which can decrease the computational effort to within only 2 or 3 times the cost of an ordinary saddle-point/MECP optimization and frequency analysis.^77−79^

Of course, in order to make useful predictions and obtain agreement with experiment, we must also consider the reliability of the underlying potentials. In typical applications, the accuracy of our calculations is limited by the electronic-structure methodology, rather than the instanton approximation itself. This is in contrast to rate theories based on separable tunneling approximations, which can lead to orders of magnitude errors and unphysical behavior even when combined with accurate electronic structure.^58,66^ Instanton theory offers a good balance of rigor with efficiency, allowing the combination of state-of-the-art multireference electronic-structure methods with a reliable description of tunneling.

The validity of the instanton approach relies on the accuracy of the steepest-descent approximations in both position and time. The approximation in time is related to the assumptions of transition-state theory in that it takes only short-time information and neglects recrossing, nuclear interference, and recoherence. The approximation in position is equivalent to a local harmonic expansion of the potentials at each point along the instanton path and the replacement of Δ by a constant. The dominant anharmonic contribution to the action is of course already included *along* the tunneling pathway, but in order to improve upon this and additionally include anharmonic effects in the fluctuations *around* the path, it is possible to obtain a perturbatively corrected instanton theory based on third- and fourth-order derivatives of the potential.^80^ However, the instanton approach (even with the perturbative correction) is not able to treat liquid systems explicitly; here one requires path-integral sampling methods instead.^43,44^ Such methods are not as strongly connected to the instanton as one would like^81^ and are only directly applicable to the normal regime.^59^ A new frontier of theoretical development is thus to develop improved path-integral sampling methods applicable to liquids, in particular for the inverted regime. Based on the connection between quantum transition-state theories^82,83^ and semiclassical instanton theory in the Born–Oppenheimer regime,^12,84^ there have been attempts to obtain a nonadiabatic rate theory connected to the golden-rule instanton.^81,85^ Like instanton theory, these methods are also based on imaginary-time path integrals and do not capture real-time dynamical behavior. Even more powerful adiabatic rate theories are based on ring-polymer molecular dynamics (RPMD),^47,86^ which also has a strong connection to instanton theory.^12^ The dream is to obtain a nonadiabatic version of RPMD^87−89^ which reduces to the nonadiabatic instanton in appropriate limits.

In this work, we have discussed applications only to spin-crossover reactions. However, Fermi’s golden rule is so named because of its broad applicability to many scientific problems of interest. In particular, electron transfers, proton-coupled electron transfers, radiationless transitions, photodissociation, electronic absorption and emission spectroscopy,^24^ and other light–matter interactions including those in polaritonic chemistry^90^ can be studied in the same way. Large tunneling enhancements have also been predicted in photosensitization reactions,^91,92^ and instanton theory is also being developed for this direction.^93^ In bridge-mediated electron transfers, there are three (or more) electronic states, and the instanton approach can be generalized straightforwardly to treat the multiple hops.^40^ Some nonadiabatic reactions occur through a conical intersection, where Δ is manifestly not a constant. In recent work, we have shown how to extend the method to treat this problem and capture the geometric-phase effect when the instanton winds around the conical intersection.^94^ In order to treat nonadiabatic reactions in which the spin state does not change, we will have to construct diabatic states, as standard electronic-structure methods return results in the adiabatic representation. This is thankfully possible due to the fact that the instanton trajectory is simply a one-dimensional line (even if it is embedded in a high-dimensional space).^95^

One might ask whether the approximation that lies behind Fermi’s golden rule is always valid. In organic chemistry, most spin–orbit couplings are small enough that it holds, but in order to confirm this and to tackle heavier elements with larger couplings, it is necessary to know what the next-order term would be. We have thus derived a rigorous fourth-order rate theory which postprocesses data obtained by the ordinary instanton optimization to calculate the Δ^4^ contribution beyond the golden rule.^96^ This work shows that there are multiple fourth-order mechanisms, some of which enhance the rate via virtual excitations and others which reduce the rate due to recrossing. To go even further and obtain a method valid for the whole range of Δ, a nonperturbative nonadiabatic instanton approach has been developed, which goes beyond previous attempts^39,97,98^ to capture the correct behavior from the golden-rule limit with weak Δ to the adiabatic Born–Oppenheimer limit with strong Δ.^99,100^

Instanton theory has come a long way since its origins in adiabatic tunneling processes and has shown itself to be a powerful formalism which can be adapted to a wide range of different scenarios including nonadiabatic chemical reactions. In our research group we are working on many further extensions of the approach and expect to be able to present applications of these new methods to realistic systems in the near future.

## Footnotes

a Here, we implicitly assume that the reactants are in thermal equilibrium and that the reaction follows an incoherent decay with a phenomenological rate constant. This is commonly the case for reactions with a significant barrier, as long as the product has a high enough density of states.

b In the case of continuum states, one replaces the sums by integrals.

c For spin crossovers, this additionally includes a sum over the different spin–orbit coupling components.^21^

d In liquids there would be an infinite number of periodic orbits with overlapping distributions. For this reason, the steepest-descent approximation inherent to the instanton approach is not directly applicable to molecules in solution, although solvent effects can be included as an effective spectral density.^56^

e As consecutive steepest-descent approximations are equivalent to one multidimensional steepest-descent integration, one could instead perform both the time and position integrals simultaneously (possibly even together with the semiclassical approximation of the propagator). This results in equivalent formulations, which we have presented in previous work.^22,24^ However, here we choose to perform the integrals separately to present a more pedagogical derivation. Either way, the final semiclassical result is an asymptotic approximation which becomes exact as *ℏ* → 0.

f At lower energies, the relative magnitudes of *W* cannot be determined by such a simple argument and the result is specific to each reaction.

g Note that this zero mode is not related to the permutational mode of adiabatic instantons. Here, the infinite result is not due to an error of the steepest-descent approximation but also occurs when calculating the correlation function exactly.

h This is relative to the NA-TST result. The ratio of the instanton prediction to the fully classical golden-rule rate is even larger at about 1000.
